# Flower Development of Heterodichogamous *Juglans mandshurica* (Juglandaceae)

**DOI:** 10.3389/fpls.2021.541163

**Published:** 2021-03-30

**Authors:** Lijie Zhang, Chong Guo, Xiujun Lu, Xiaomei Sun, Chunping Liu, Qiang Zhou, Jifeng Deng

**Affiliations:** ^1^College of Forestry, Shenyang Agricultural University, Shenyang, China; ^2^Key Laboratory of Forest Tree Genetics and Breeding of Liaoning Province, Shenyang, China; ^3^Liaoning Forestry and Grassland Administration, Shenyang, China

**Keywords:** reproductive development, protandry, protogyny, heterodichogamy, *Juglans mandshurica* maxim

## Abstract

*Juglans mandshurica* is a monoecious heterodichogamous species with protogynous and protandrous mating strategies that occur at a 1:1 ratio and are randomly distributed in the population. The inconsistent male and female flowering periods of the same mating type result in an imbalance of the ratio of male and female flowers, contributing to the low yield of this species. However, little more is known about its floral development. Following three consecutive years of observations, histological analysis, and scanning electron microscopy, we found that the morphological and anatomical development of the male and female flowers were synchronous. The male floral morphological development of *J*. *mandshurica* was divided into seven phases, while that of the female flower was nine. Four stages were shared between the male and female flower’s anatomical development. Our findings indicate that there was minimal overlap between sexual functions within the same mating type, guaranteeing synchronization, mutual non-interference, outcrossing, and avoidance of self-fertilization. These results provide a theoretical basis for the improvement of fruit yield and quality through the reasonable allocation of protogynous and protandrous individuals in a population, and for artificial pollination control. Further, these findings lay a foundation for further research on the genetic mechanisms and environmental effects on flower development of heterodichogamous *J*. *mandshurica*.

## Introduction

Heterodichogamy is a transitional mating type between hermaphroditic and dioecious plants (Bai et al., 2006) that contains two mating types, protogynous (female function before male) and protandrous (male function before female) (Endress and Lorence, 2004; Stahl et al., 2019). These two mating types can co-occur within one population, and the ratio of the two types is nearly always 1:1 (Teichert et al., 2011; Wang et al., 2012; Fukuhara and Tokumaru, 2014; Renner, 2014). Heterodichogamous plants include species of *Acer* (Field and Barrett, 2012), *Alpinia* (Li et al., 2001), *Carya* (McCarthy and Quinn, 1992), *Corylus* (Muller, 1875), *Grayia* (Pendleton et al., 1988), *Hernandia* (Endress and Lorence, 2004), *Persea americana* (Stahl et al., 2019), and *Juglans* (Kimura et al., 2003). Notably, although some *Acer* species have bisexual flowers, the flowers are functionally unisexual or monoecious (Jacobs and Lersten, 1994).

Monoecious plant species have separate male and female flowers on the same plants and can therefore undergo self-fertilization when the pollen of staminate flowers contacts the stigma of pistillate flowers, barring other defenses against selfing (Mao et al., 2017; Avalos et al., 2019; Hildesheim et al., 2019). Heterodichogamy, which is dispersed and reported in 20 genera from 13 families of flowering plants, is one of the evolutionary pathways from monoecy to dioecy, heterodichogamous populations usually contain two sexual morphs, protogynous and protandrous, which are reciprocal (Meng et al., 2009; Liu et al., 2016). Hermaphroditism brings about the risk of pollination interference, pollen wastage, etc., but does not prevent it. Other mechanisms like heterodichogamy have evolved to prevent these effects (Ye et al., 2019). *Juglans mandshurica* Maxim (*J. mandshurica*) is a monoecious heterodichogamous species also referred to as *Carya cathayensis* and commonly known as hickory. *J. mandshurica* is one of the “three hardwood and broad-leaved tree species” (along with *Fraxinus mandshurica* and *Phellodendron amurense*) known to occur in the Xiaoxinganling Mountains, Wanda Mountains, Changbai Mountains, the eastern mountainous areas of Liaoning province, China; it has also been documented in Russia’s Far East, in North Korea, and in Japan (Wang et al., 2008; Zhao and Woeste, 2011; Zhang et al., 2012; Zhao et al., 2014; Hu et al., 2016; Jin et al., 2016). *J. mandshurica* is a tertiary relict species and is a national class II and class III protected rare plant and endangered tree species in China (Chen et al., 2014). *J. mandshurica* is an economically important woody plant (Ros and Mataix, 2006; Hasan et al., 2011; Salimi et al., 2012; Vinson and Cai, 2012; Zhao et al., 2014), but existing *J. mandshurica* resources are distributed primarily in natural forests, which are under threat due to excessive logging and imbalances in harvesting and breeding (Guo et al., 2015). At present, yields of *J. mandshurica* walnut fruit are in sharp decline, and timber yields account for less than 1% of the total timber output of the eastern mountainous areas of Liaoning, China. The developmental asynchronization of flowers in protogynous and protandrous mating types in *J. mandshurica* could prevent selfing and increase the probability of outcrossing, but could also result in an imbalance in the ratio of male and female flowers, thereby affecting yields. Although *J. mandshurica* has been extensively studied, research has thus far primarily focused on its pharmacological properties (Carvalho et al., 2010; Sharma et al., 2013; Xu et al., 2013) and population dynamics (Wang et al., 2015). Few reports have studied the reproductive biology of heterodichogamous *J. mandshurica* in Eastern Liaoning Province, China, where *J. mandshurica* naturally occurs in the greatest density. Therefore, in the present study, the morphology and anatomical structure of flowers at each phenological growth stage were observed and investigated continuously. This research could provide a scientific basis for further studies on the molecular regulatory mechanisms governing flower development in heterodichogamous plants.

## Materials and Methods

### Tree Species

**FIGURE 1:**
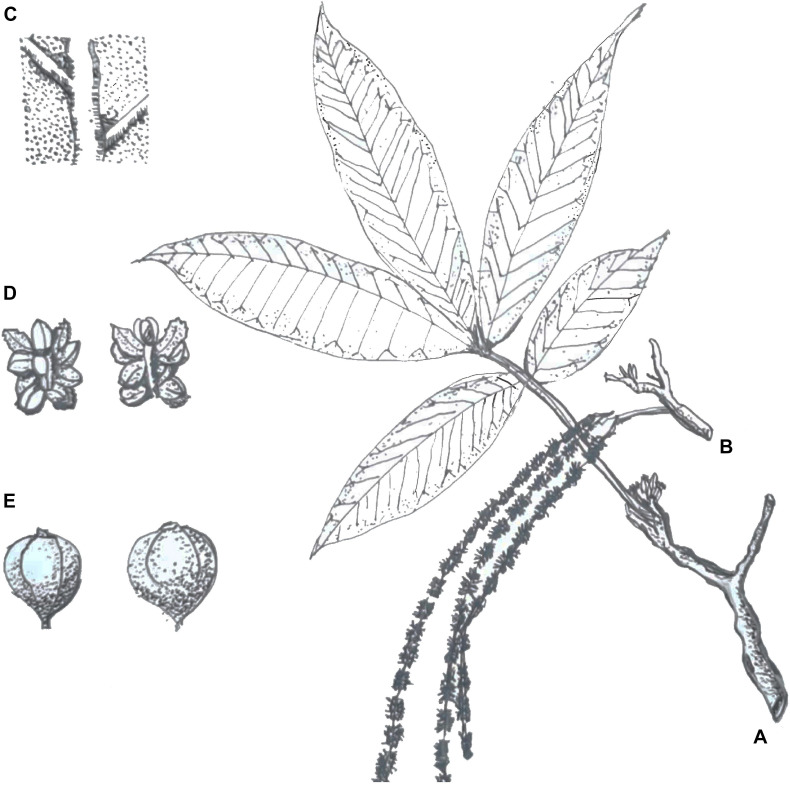
Botanical sketches of *Juglans mandshurica* Maxim. Panel **(A)** shows the strip (e.g., spikelets and leaves), panel **(B)** shows catkins, panel **(C)** shows the abaxial surface of the leaf, panel **(D)** shows a staminate flower, and panel **(E)** shows the fruit (redrew from the artist: Taili Zhang, born in Jin Zhou, China 1938).

*Juglans mandshurica* is a monoecious and deciduous species of tree that is predominantly wind-pollinated. The tree reaches heights of 20 m with extended branches, an oblate crown shape, and gray bark (Bai et al., 2006). *J. mandshurica* commences flowering in early spring. The young shoot is covered with short hairs. Odd-pinnate leaves on sprouts can reach up to 80 cm, and the petiole is 9–14 cm long, with 15–23 leaflets of 6–17 cm long and 2–7 cm wide. The staminate inflorescences are pendulous catkins 9–20 cm in length with 4–10 male flowers per catkin. The flowers have 12 stamens, and the anthers are about 1 mm long. The pistillate inflorescences are vertical spikelets 3–5 cm in length with 10–18 flowers. The gynoecium has a single ovule and two feathery stigmas (Figure 1).

### Study Site and Plant Material Selection

The study was conducted in the Dichegou experimental forest farm (longitude 124 20′ 06″–125 28′ 58″ E, latitude 41 47′ 52″–42 28′ 25″ N), which is located in Wandianzi town, QingYuan Manchu Autonomous County, Fushun City, Liaoning Province, China. The area has a mid-temperate continental monsoon climate, with cold and dry winters, hot and rainy summers, an annual average temperature of 6.6°C, an annual accumulated temperature of 2,700–3,200°C, and a frost-free period of 120–125 days. A total of 200 *J. mandshurica* sample trees (100 protogynous and 100 protandrous) were selected at the study site, and the dates of onset and termination of male and female flowering were recorded for each individual tree from 2017 to 2019. The periods of male and female flowering were determined as described by Bai et al. (2006).

### Histological Analysis

Male and female flower buds were sampled every 3 days from March to June of 2019 (in accordance with tree sap flow activities). The samples were vacuum filtered with FAA fixative for 1 h, and dehydrated sequentially with 50, 70, 85, 95, and 100% ethanol alcohol for 2 h each at room temperature. The samples were then transferred to a mixed solution of 1:1 xylene and anhydrous alcohol for 1 h, after which 20 mL of pure xylene was added for approximately 2 h to make the samples visible. The samples were then placed in a mixed solution of ethanol and xylene at ratios of 1:3, 1:1, and 3:1 ethanol: xylene for 1 h each. This process was carried out in a Thermal Chamber (Lisun Electronics Co., Ltd., Shanghai, China) at 3°C above the melting point of paraffin wax (approximately 40°C). The samples were subsequently placed in dissolved pure paraffin wax for 30 min, and the process was repeated three times. Paraffin fixing and section slicing were performed using a Leica CM1850 V2.0 (Leica, Germany). The thickness of the wax slices was approximately 8 μm. Sections were double-stained according to Sass’s method (Ruzin, 1999). The sections were sequentially placed into the xylene I, xylene II, anhydrous ethanol I, anhydrous ethanol II, 95% alcohol, 90% alcohol, 80% alcohol, and 70% alcohol for 20, 20, 10, 10, 5, 5, 5, and 5 min, respectively, and were washed with distilled water after being thoroughly dried. The sections were then placed into a 1% safranine dye solution and dyed for 1–2 h. Tap water was used to remove any excess dye solution. Next, 50, 70, and 80% alcohol was added to the sections for 3 min each for decoloration, following which the sections were dyed in 0.5% solid green dye solution for 30–60 s. Decoloration was carried out in anhydrous ethanol I for 30 s and anhydrous ethanol II for 1 min. Finally, the sections were dried in an oven at 60°C and placed in xylene solution at ratios of 1:3 ethanol: xylenef for 5 min. The sections were sealed with Canada balsam and photographed under SZX10 stereo and BX53 microscopes (Olympus, Japan).

### Scanning Electron Microscopy

All of the sampled buds were dissected by removing their scales and leaves and fixed with 4 glutaraldehyde for 12 h, rinsed with 0.1 molL^–1^ of phosphoric acid buffer for 1 h, dehydrated with 30, 50, 70, 85, and 90% ethanol alcohol for 15 min each, and dehydrated with 100% absolute ethyl alcohol for 30 min. After freeze-drying, the materials were critical-point dried using liquid CO_2_ (Emitech K850, United Kingdom). Samples were placed on a display board and glued. The treated materials were sputter-coated with gold (Hitachi E-1010, Japan) and then observed using an S4800 scanning electron microscope (Hitachi, Japan).

## Results

### Flowering Phenology

**FIGURE 2:**
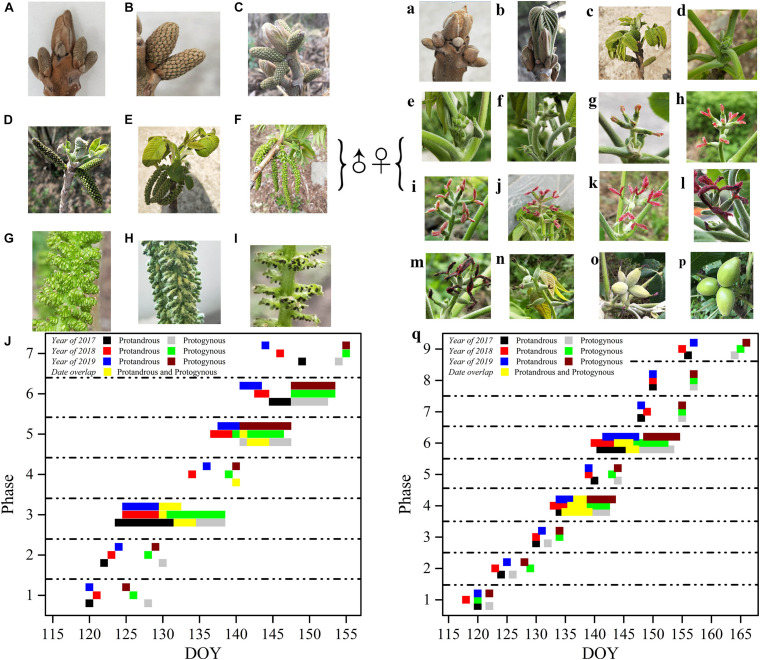
Flowering sequence of protogynous and protandrous *Juglans mandshurica* trees. Capital letters represent male flowers, lowercase letters represent female flowers; ♀: male flower, ♂: female flower. Panel **(A)** represents dormancy of bud in winter, **(B)** phase 1: differentiation of scale, **(C)** phase 2: formation of catkin, **(D)** phase 3: elongation of catkin, **(E)** phase 4: formation of anther, **(F)** phase 5: mature anther, **(G)** represents enlargement of mature anther, **(H)** phase 6: pollen dispersal of catkin, and **(I)** phase 7: withering of catkins. Panel **(a)** presents dormancy of bud in winter, **(b)** phase 1: bract breaking, **(c)** phase 2: leaf spreading, **(d,e)** phase 3: formation of bud and bud breaking, **(f)** phase 4: elongation of bract, **(g,h,j,k)** phase 5: flowering early period (protandry), flowering peak period (protandry), flowering early period (protogyny), and flowering peak period (protogyny), **(i,l)** phase 6: flower pollination (protandry) and flower pollination (protogyny), **(m)** phase 7: withering of spikelets, **(n)** phase 8: enlargement of ovary, **(o)** phase 9: fruit setting, **(p)** represents stone fruit; and **(J,q)** represent flowering period of caktins and spikelets in 2017, 2018, and 2019, respectively.

The morphological development of male *J*. *mandshurica* flowers was divided into seven phases, while the female flower development was divided into nine phases (Figure 2). The male flower buds displayed rust yellow coloration and pilose surfaces with 5–7 mm long hairs. The catkins were 9–20 cm long with a pilose axis. The male flowers had short floral shoots and two bracteoles subtending the petal. The anthers turned from green (immature) to black (after dehiscence), and finally fell off of the shoots. The female bud surface was pilose with 9–15 mm hairs, and the bud body was brown. In the early stages of development, it was difficult to distinguish the female floral bud from the leaf bud until the air temperature gradually rose and the buds broke, revealing odd-pinnate leaves. Before stage 4 (opening of the bifid stigma to form inverted splayed petaloids), there was no obvious morphological difference between the female flower buds in protogynous and protandrous types, except for the flowering time. At stage 5, such differences became more obvious due to color. The protandrous male flower development lasted approximately 25 days (the starting time was DOY 115, when the sap flow begins, inflorescences started to flower after 1-year development, and new shoots were generated, this time refers to day 0), while the protogynous male flower development lasted approximately 31 days. The female flower development of the protogynous type lasted approximately 37 days, while the protandrous female flower development lasted approximately 45 days.

### Flower Development Stages

**FIGURE 3:**
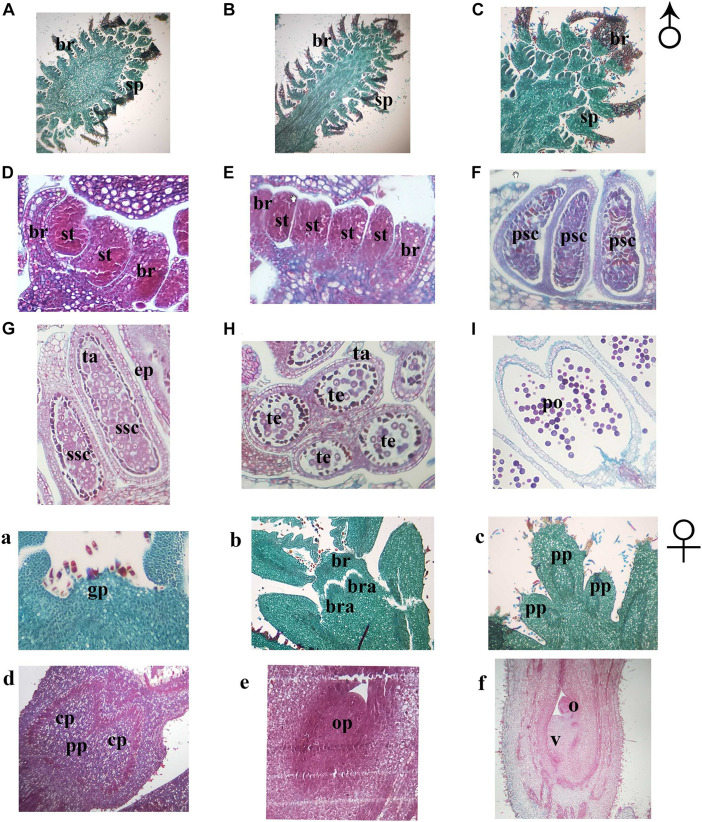
Sections of flower bud tissue from protogynous and protandrous *Juglans mandshurica* trees. Panel **(A)** presents initial differentiation of floral bud, panel **(B)** presents initial formation of stamen, panel **(C)** presents catkins, panel **(D)** presents initial formation of anthers, panel **(E)** presents primary sporogenous cell, panel **(F)** presents secondary sporogenous cell, panel **(G)** presents internal structure of anther, panel **(H)** presents pollen sac, panel **(I)** presents pollen grain. Panel **(a)** presents initial differentiation of floral bud, panel **(b)** presents differentiation of inflorescence primordia, panel **(c)** presents involucral primordia, panel **(d)** presents initial formation of primordial ovule, panel **(e)** presents primordial ovule, and panel **(f)** presents formation of ovule and ovary. an: immature anther; br, bract; bra, bracteoles; cp, carpellary primordia; ep, epidermal layer; fa, anther after disperse pollen; gp, growth cone; ma, mature anther; o, ovule; op, ovule primordia; po, pollen; pp, pistil primordia; psc, primary sporogenous cell; s, stamen; sp, stamen primordium; ssc, secondary sporogenous cell; ta, tapetum; and v, ovary.

In order to further understand the developmental characteristics of male and female flowers, flower development was then divided into several stages by key morphological events and developmental order of flower organs with the results of the histocytological changes (Figure 3). In both mating types, the development of both the male and female flowers of *J. mandshurica* was divided into four stages. For male flowers, the stages included the initial differentiation of the male flower bud (related to phase 1), formation and development of stamen primordia, formation and development of catkins (related to phases 2 and 3), and the formation of anthers and pollen grains (related to phases 4 and 5); for female flowers, stages included initial differentiation of the female flower bud (related to phase 3), formation and development of pistil primordia, formation and development of bract and sepal primordia (related to phase 4), and formation and development of ovule primordia (related to phase 5). The differentiation and development of the male flowers of protandrous trees was earlier than that of protogynous trees, while the differentiation of male flowers of protogynous trees was about 3–5 days later than that of protandrous trees. The paraffin section micrographs could reflect the different flower development stages well.

**FIGURE 4:**
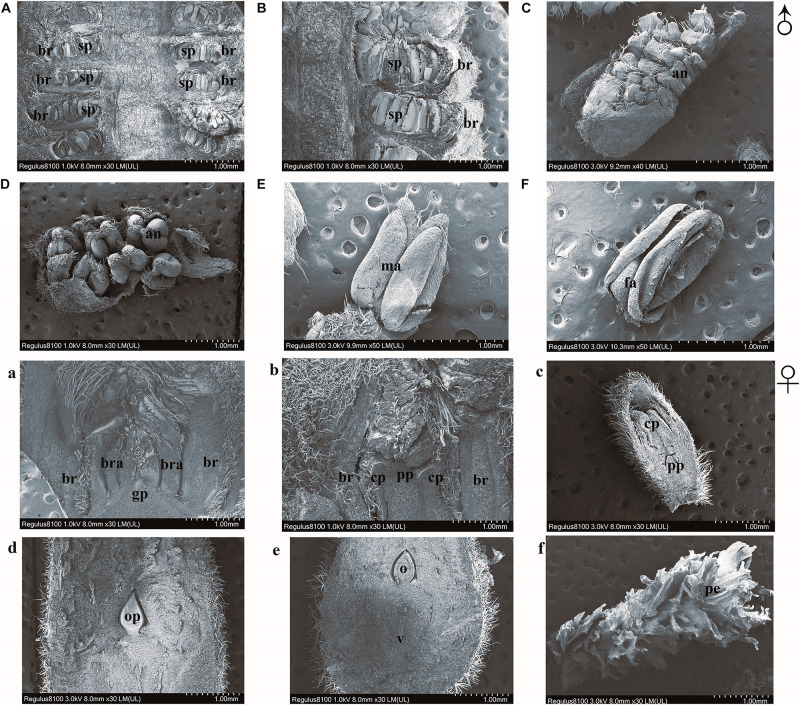
Scanning electron micrographs of flower buds of *Juglans mandshurica*. Panel **(A)** shows dormancy of bud in winter, panel **(B)** shows germination of bud, panel **(C)** shows elongation of catkin, panel **(D)** shows formation of anther, panel **(E)** shows pollen dispersal of catkin, panel **(F)** shows withering of catkins, panel **(a)** shows dormancy of bud in winter, panel **(b)** shows germination of bud, panel **(c)** shows formation of bud, panel **(d)** shows flowering period, panel **(e)** shows enlargement of ovary phase, and panel **(f)** shows a petaloid.

To probe flower buds of *J*. *mandshurica* into more visible way. Scanning electron micrographs were taken and three-dimensional images were obtained. Stamen primordia can be seen inside the dormant buds in winter (Figure 4A). The bract primordia started to grow and it can be seen on the outer edge of the stamen primordia (Figure 4B) and catkins began to elongate, and individual stamens then appeared (Figure 4C). At this time, the anther gradually formed (Figure 4D). When anthers became mature, they broke and dispersed the pollen (Figure 4E) until gradually withering (Figure 4F). Bud primordia were clearly seen during winter dormancy (Figure 4a). The bud primordia were flattened, expanded, and raised in three locations directly after germination of the bud (Figure 4b). The middle spot developed into a primordial ovule, and the other two spots became petals (Figure 4f). Figure 4c presents the pistil primordium; its upper part developed into a stigma (Figure 4d), while its lower part developed into an ovary, which surrounded the ovule and then expanded (Figure 4e), completing the fertilization process.

## Discussion

Most species of tree in the genus *Juglans* are self-pollinated, but our findings strongly indicate that *J. mandshurica* cross-pollinates, a finding consistent with the flowering phenological characteristics of *J. mandshurica* reported by Bai et al. (2007). In addition, research conducted on *J. mandshurica* indicated that the sexual functions of the two mating types were synchronous and reciprocal within a given population. Therefore, heterodichogamy in *J. mandshurica* may represent an effective strategy for reducing self-pollination, but if the strategy could increase the pollination efficacy, pollen supplementation and fruit production needed to be tested in the future. However, some studies have shown that outcrossing may shorten the life span of flowers as compared to selfing (Devlin and Stephenson, 1984; Richardson and Stephenson, 1989; Gregg, 1991; Aizen, 1993). Further research building upon the results of the current work will aid in confirming the efficacy of heterodichogamy and outcrossing as reproductive strategies in *J. mandshurica*.

Adverse climatic conditions at the time of flowering severely hinder yields and may lead to the failure of flower development (Borghi et al., 2019). Nowadays, decline of forests has accelerated in recent decades at the regional and continental scales, as air temperature is continuously rising and models predict a sharp decline in the strength of the terrestrial carbon sink in the next century (Adams et al., 2013). Even if precipitation remains same to the historical levels, continuous warming-associated extremes raise the possibilities of dieback affecting the metabolism and indirect effects on the flower physiological responses, flower metabolic responses, and flowering stages (McDowell et al., 2013). While, to make clear of mechanism of flowering of plants may be dramatic and difficult to predict than is currently assumed, because of the numerous contributing factors (Anderson et al., 2012). Especially in northern China, where human activities and natural hazards like deforestation, over-cultivation, urbanization, wildland fire, has resulted in the degradation of these regions and further threatened the *J. mandshurica* tree species (Liu et al., 2016). Therefore, more detailed work should focus on the expression of genes associated with known pathways in response to warming and drying trends, downstream signaling from environmental stress responses and flower development and other mechanisms explaining the floral development of male and female flowers in *J*. *mandshurica* individually, locally, and regionally, which can toward a common understanding of these processes to the benefit of ecology and society.

## Conclusion

The pattern of flower phenology was observed and thoroughly investigated in 200 individuals *J. mandshurica* trees during 2017–2019. Minimal overlap was observed between the two sexual functions within individual trees, and the flowering periods of protogynous and protandrous mating types were reciprocal and synchronous, thus promoting outcrossing. The distinctive developmental stages of the male and female floral morphology were classified and revealed in detail.

## Data Availability Statement

The original contributions presented in the study are included in the article/supplementary material, further inquiries can be directed to the corresponding author.

## Author Contributions

All authors commented on the manuscript at all stages. JD and LZ conceived and designed the study. CG, XL, XS, CL, QZ, JD, and LZ contributed the materials and analysis tools and contributed to the data analysis and manuscript preparation.

## Conflict of Interest

The authors declare that the research was conducted in the absence of any commercial or financial relationships that could be construed as a potential conflict of interest.
